# Coronary revascularization after surgical aortic valve replacement

**DOI:** 10.1016/j.xjon.2020.05.005

**Published:** 2020-05-29

**Authors:** Mevlüt Çelik, Andras P. Durko, Stuart J. Head, Edris A.F. Mahtab, Nicolas M. van Mieghem, Paul A. Cummins, Arie P. Kappetein, Ad.J.J.C. Bogers

**Affiliations:** a Department of Cardiothoracic Surgery, Erasmus University Medical Center, Rotterdam, The Netherlands; b Medtronic, Maastricht, The Netherlands; c Department of Cardiology, Erasmus University Medical Center, Rotterdam, The Netherlands

**Keywords:** aortic stenosis, aortic valve replacement, transcatheter, coronary artery bypass grafting, percutaneous coronary intervention, AS, aortic valve stenosis, CABG, coronary artery bypass grafting, CAD, coronary artery disease, LVEF, left ventricular ejection fraction, PCI, percutaneous coronary intervention, SAVR, surgical aortic valve replacement, TAVR, transcatheter aortic valve replacemen

## Abstract

**Objective:**

It remains unclear how often coronary revascularization is necessary after aortic valve interventions, either by surgical aortic valve replacement (SAVR) or transcatheter aortic valve replacement. However, these data are relevant for treatment and prosthesis choice. The authors sought to analyze the incidence and characteristics of coronary revascularization after SAVR during follow-up.

**Methods:**

Of 2256 patients undergoing isolated SAVR between 1987 and 2015, 420 patients (mean age 56.9 ± 15.5 years, 66.9% male) were followed at the Erasmus Medical Center. Incidence, predictors, and characteristics of coronary revascularization were analyzed. Cumulative incidence of revascularization was assessed using a competing risk approach.

**Results:**

Mean follow-up after SAVR was 17.2 years (total of 4541 patient-years). A total of 24 patients underwent 28 procedures of revascularization. The cumulative incidence of revascularization after SAVR was 0.5%, 2.2%, 4.1%, and 6.9% at 1, 5, 10, and 20 years, respectively. The linearized rate of revascularization was 6.2 per 1000 patient-years. Percutaneous coronary intervention was the most common revascularization method (64%; N = 18/28). Revascularization before SAVR (N = 36/420; of whom 27 percutaneous coronary intervention) was an independent predictor of revascularization during follow-up (hazard ratio, 6.6; 95% confidence interval, 2.6-17.1; *P* < .001).

**Conclusions:**

After SAVR, the rate of coronary revascularization was 6.9% (N = 24/420) at 20-year follow-up. Patients were at particular risk if they had undergone previous revascularization before SAVR. These data may furthermore be relevant to the transcatheter aortic valve replacement population.

## Graphical abstract

**Figure:**
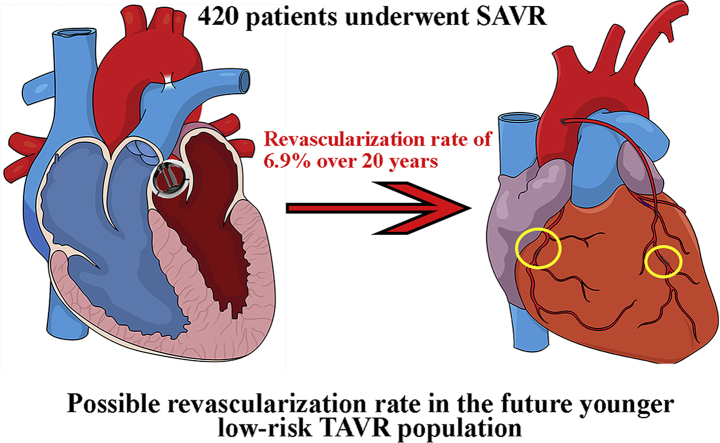

**Figure:**
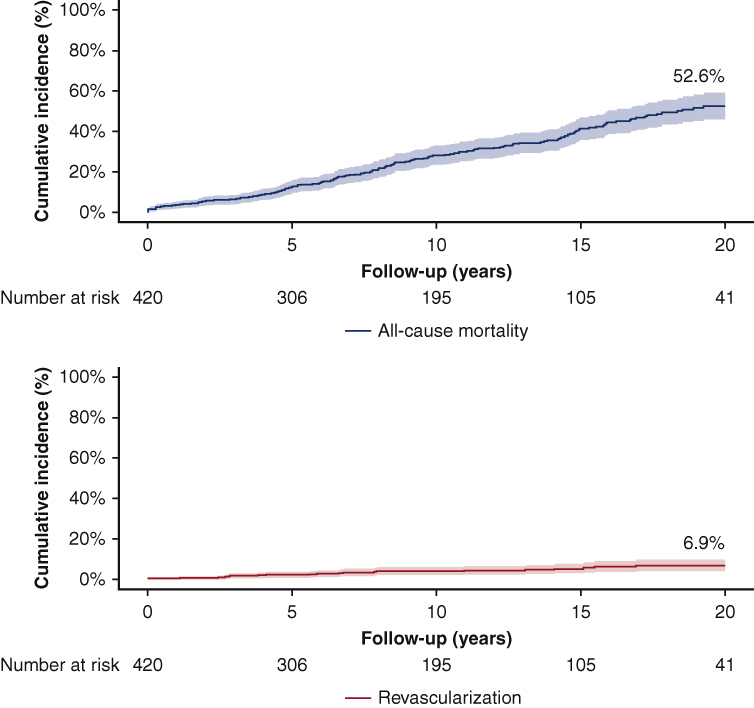
Competing risk cumulative incidences of mortality and revascularization; 20-year follow-up.

**Central Message**

In a large SAVR cohort, the rate of coronary revascularization was 6.9% after 20-year follow-up. Previous revascularization was an independent predictor of revascularization after SAVR during follow-up.

**Perspective**

Coronary revascularization rates after SAVR can be used to predict the need for revascularization after TAVR, should TAVR further expand into younger, lower-risk populations. Dedicated studies are required to address the incidence, predictors, and feasibility of revascularization after TAVR.

See Commentaries on pages 102 and 104.

Transcatheter aortic valve replacement (TAVR) is now recommended for patients with severe aortic valve stenosis (AS) at intermediate and high surgical risk,^1^^,^^2^ adding more evidence to the already ongoing increase in the number of performed TAVR procedures in North America and Europe.^3^^,^^4^ Recent trials that included low-risk patients have reported noninferiority or even superiority of TAVR versus surgical aortic valve replacement (SAVR).^5^^,^^6^

Reports have suggested that access to the coronary arteries may be difficult to establish after TAVR as a result of the positioning of the transcatheter valve.^7^ When indication expands toward low-risk patients, who often are younger, the need for coronary revascularization after TAVR may increase. However, due to the advanced age and presence of multiple comorbidities of patients in current TAVR trials and the relatively short-term follow-up available, the incidence of coronary revascularization has been difficult to determine. The probability of coronary revascularization after TAVR may increase in patients with longer life expectancies, with potential implications for procedure and prosthesis choices.

SAVR has been the standard of care for AS over the past 50 years. Therefore, long-term follow-up is available to determine the incidence of coronary revascularization after SAVR in low-risk patients. Since the historical SAVR patient population overlaps with current and future TAVR patient populations, data of revascularization after SAVR can provide insights into determining which surgical or transcatheter prostheses may be more appropriate in specific patients. The aim of this study was to assess the incidence and risk factors of coronary revascularization during long-term follow-up after SAVR.

## Methods

### Study Design

**Figure 1:**
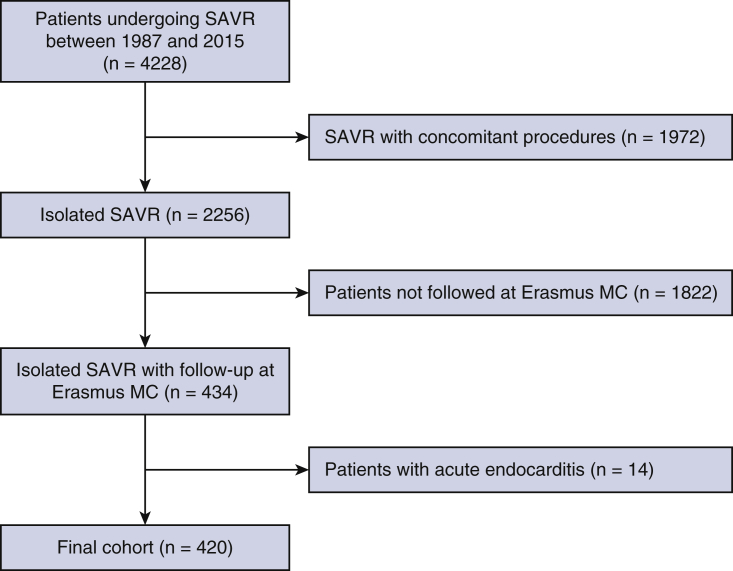
Flowchart of patient inclusion. A total of 4228 patients underwent SAVR at the Erasmus MC between 1987 and 2015, of whom a total of 420 patients were eligible for the study. *SAVR*, Surgical aortic valve replacement.

This observational, retrospective study consisted of adult (≥18 years) patients who underwent isolated SAVR with a mechanical or bioprosthetic valve between 1987 and 2015 at the Erasmus Medical Center (Erasmus MC), Rotterdam, The Netherlands. To ensure that all coronary revascularization procedures during follow-up were captured, only patients followed up at the outpatient clinic of the Erasmus MC were included in this study (Figure 1). Patients undergoing concomitant procedures or with active endocarditis were excluded. Coronary artery disease (CAD) was routinely assessed before SAVR by coronary angiography, and patients with CAD underwent concomitant coronary artery bypass grafting (CABG) according to the recommendations of clinical guidelines in use at the time of surgery and were excluded.

The study was approved by the local institutional review board, and patient-informed consent was waived. All the authors assured for the validity of the data and adherence to the protocol.

### Data Collection and Follow-up

Baseline patient and procedural characteristics were collected from electronic medical records. Survival status was obtained through the National Death Registry.

After SAVR, patients returned to their referring cardiologist at Erasmus MC for routine, regular outpatient clinic visits at 3 and 6 months postoperatively and (bi-)annually thereafter. If CAD was diagnosed and revascularization was deemed necessary, patients underwent either percutaneous coronary intervention (PCI) or CABG at the Erasmus MC.

### End Points and Definitions

The primary end point was coronary revascularization either by PCI or CABG. SAVR within 24 hours of establishing the indication was classified as urgent, between 24 hours and 3 days as semi-elective, and after 3 days as elective. Left ventricular function was classified as normal if the left ventricular ejection fraction (LVEF) was >50%, as mildly reduced if the LVEF was 40% to 50%, as moderately reduced if the LVEF was 30% to 40%, and as severely reduced if the LVEF was less than 30%, as measured or estimated by a trained echocardiographer.

### Statistical Analyses

Discrete variables are presented as numbers, percentages or proportions, and compared with either the χ^2^ test or the Fisher exact test, where appropriate. Continuous variables are presented as means ± standard deviation or median with the interquartile range if there was evidence of skewed data according to the Kolmogorov–Smirnov test, and compared with either the 2-sample *t* test or Wilcoxon rank-sum test, where appropriate.

Probabilities of the occurrence of revascularization and mortality were visualized using cumulative incidence curves with their according 95% confidence intervals. The cumulative incidence based on Kaplan–Meier estimates does not reflect the competing risk of death and the occurrence of revascularization and therefore overestimate the remaining lifetime risk of revascularization when the competing risk is high.^8^ To account for this overestimation, competing risk survival analysis was performed by means of nonparametric methods using the cumulative incidence competing risk method.^9^^,^^10^ Post-hoc subgroup analyses were performed according to whether revascularization had taken place before the SAVR procedure, age at time of SAVR (aged <65 or ≥65 years), history of hypercholesterolemia, history of diabetes mellitus, indication of SAVR (AS, aortic valve regurgitation, or combined disease), and type of implanted valve (mechanical or bioprosthetic). Competing risk survival analyses in subgroups were compared with the Fine and Gray test.^11^ Furthermore, the linearized rate of revascularization was calculated per 1000 patient-years of follow-up.

Predictors of revascularization after SAVR were identified in a Cox proportional hazards model. Significant variables on univariable analyses were included in a multivariable Cox proportional hazards model. Data analyses were performed using SPSS 24.0 (IBM Corp, Armonk, NY) and R software, version 3.4 (R Foundation for Statistical Computing, Vienna, Austria).

## Results

### Baseline and Procedural Characteristics

**Figure 2:**
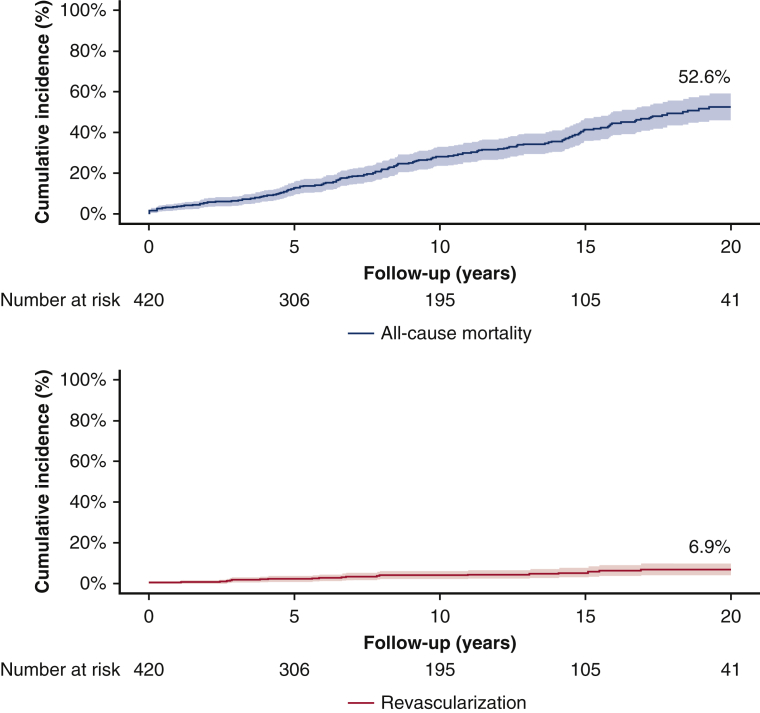
Mortality and coronary revascularization after SAVR. Competing risk cumulative incidences of mortality and coronary revascularization during 20-year follow-up according to (A) *blue line* presents the cumulative incidence of all-cause mortality competing with the risk of revascularization in our cohort and (B) *red line* presents the cumulative incidence of revascularization with either PCI or CABG competing with the risk of revascularization in our cohort. *SAVR*, Surgical aortic valve replacement; *PCI*, percutaneous coronary intervention; *CABG*, coronary artery bypass grafting.

From 4228 patients who underwent SAVR between 1987 and 2015, 420 patients underwent isolated SAVR and were followed up at the Erasmus MC and were included in this study (Figure 1). The mean age of the patients at the time of SAVR was 56.9 ± 15.5 years, and 66.9% (281/420) were male. The primary indication for SAVR was pure AS in 52.1% (219/420). A total of 8.6% (36/420) had previous revascularization. Mechanical valve prostheses were used in 66.7% (280/420). The rates of survival were 98.3%, 96.4%, 87.4%, 71.8%, 58.6%, and 47.4% at 30 days, and 1, 5, 10, 15, and 20 years of follow-up, respectively (Figure 2). Detailed baseline and procedural characteristics are provided in Table 1. Patients excluded from our study were older (66.1 ± 11.1 vs 56.9 ± 15.5 years, *P* < .001), had undergone more redo SAVR procedures (16.7% vs 4.3%, *P* < .001), more often underwent SAVR with an urgent indication (4.0% vs 0.4%, *P* < .001), and had less-frequent implantation of mechanical valve prosthesis (66.7% vs 48.0% *P* < .001) compared with the included patients. Further detailed characteristics of patients excluded from our study are provided in Table 2.

**Table 1:** Baseline and procedural characteristics

	All patients (n = 420)	No revascularization (n = 396)	Revascularization (n = 24)	*P* value
Age, y	56.9 ± 15.5 (420)	56.8 ± 15.7 (396)	58.5 ± 11.6 (24)	.592
Male sex	66.9 (281/420)	67.2 (266/396)	62.5 (15/24)	.637
Primary indication				.950
AS	52.1 (219/420)	52.3 (207/396)	50.0 (12/24)	
AR	25.5 (107/420)	25.5 (101/396)	25.0 (6/24)	
Combined AS + AR	22.4 (94/420)	22.2 (88/396)	25.0 (6/24)	
Bicuspid aortic valve	24.0 (101/420)	24.0 (95/396)	25.0 (6/24)	.910
Previous cardiac operation	28.6 (120/420)	28.8 (114/396)	25.0 (6/24)	.690
SAVR	16.7 (70/420)	16.7 (66/396)	16.7 (4/24)	>.999
CABG	2.6 (11/420)	2.3 (9/396)	8.3 (2/24)	.071
Other	9.3 (39/420)	9.3 (39/396)	0	.107
Hypertension	29.8 (125/420)	29.8 (118/396)	29.2 (7/24)	.948
Hypercholesterolemia	12.4 (52/420)	11.1 (44/396)	33.3 (8/24)	.001
Diabetes mellitus	9.3 (39/420)	8.8 (35/396)	16.7 (4/24)	.199
Arterial disease	3.6 (15/420)	3.3 (13/396)	8.3 (2/24)	.195
Peripheral	3.6 (15/420)	3.3 (13/396)	8.3 (2/24)	.195
Carotid	0.5 (2/420)	0.5 (2/396)	0	.727
Renal failure	2.6 (11/420)	2.5 (10/420)	4.2 (1/24)	.625
Previous myocardial infarction	4.3 (18/420)	4.0 (16/396)	8.3 (2/24)	.313
Previous revascularization	8.6 (36/420)	7.3 (29/396)	29.2 (7/24)	<.001
Previous PCI	6.4 (27/420)	5.6 (22/396)	20.8 (5/24)	.003
Previous CABG	2.6 (11/420)	2.3 (9/396)	8.3 (2/24)	.071
Previous decompensated heart failure	16.9 (71/420)	16.4 (65/396)	25.0 (6/24)	.276
Left ventricular function				.460
Preserved	77.6 (287/370)	77.6 (273/370)	77.8 (14/18)	
Mildly reduced	7.6 (28/370)	8.0 (28/370)	0	
Moderately reduced	9.2 (34/370)	8.8 (31/370)	16.7 (3/18)	
Severely reduced	5.7 (21/370)	5.7 (20/370)	5.6 (1/18)	
Atrial fibrillation	13.3 (56/420)	13.4 (53/396)	12.5 (3/24)	.902
Previous neurologic event	10.5 (44/420)	11.1 (44/396)	0	.084
CVA	4.8 (20/420)	5.1 (20/396)	0	.259
TIA	7.1 (30/420)	7.6 (30/396)	0	.162
COPD	8.3 (35/420)	8.3 (33/396)	8.3 (2/24)	>.999
Liver disease	1.4 (6/420)	1.5 (6/396)	0	.544
History of malignancy	8.1 (34/420)	8.1 (32/396)	8.3 (2/24)	.965
Urgency				.610
Elective	49.3 (173/351)	49.4 (165/334)	47.1 (8/17)	
Semi-elective	46.7 (164/351)	46.7 (156/334)	47.1 (8/17)	
Urgent	4.0 (14/351)	3.9 (13/334)	5.9 (1/17)	
Logistic EuroSCORE	5.7 ± 6.2 (204)	5.5 ± 6.1 (193)	8.8 ± 7.3 (11)	.085
Mechanical prosthesis	66.7 (280/420)	66.7 (264/396)	66.7 (16/24)	>.999
Year of operation				.383
1987-1994	24.5 (103/420)	23.7 (94/396)	37.5 (9/24)	
1995-2001	23.3 (98/420)	24.0 (95/396)	12.5 (3/24)	
2002-2008	26.7 (112/420)	26.8 (106/396)	25.0 (6/24)	
2009-2015	25.5 (107/420)	25.5 (101/396)	25.0 (6/24)	

Data are presented as % (n/N) and mean ± standard deviation or median (interquartile range). *AS*, Aortic valve stenosis; *AR*, aortic regurgitation, *SAVR*, surgical aortic valve replacement; *CABG*, coronary artery bypass grafting; *PCI*, percutaneous coronary intervention; *CVA*, cerebrovascular accident; *TIA*, transient ischemic attack; *COPD*, chronic obstructive pulmonary disease; *EuroSCORE*, European System for Cardiac Operative Risk Evaluation.

**Table 2:** Baseline and procedural characteristics

	Patient followed-up in Erasmus MC	Patient not followed-up in Erasmus MC	*P* value
Age, y	56.9 ± 15.5 (420)	66.1 ± 11.1 (1782)	<.001
Male sex	66.9 (281/420)	57.4 (1023/1782)	<.001
Primary indication			
AS	52.1 (219/420)	69.8 (1243/1782)	<.001
AR	25.5 (107/420)	12.7 (226/1782)	<.001
Combined AS + AR	22.4 (94/420)	17.3 (308/1782)	.015
Bicuspid aortic valve	24.0 (101/420)	19.2 (343/1782)	.027
Previous cardiac operation	28.6 (120/420)	8.6 (154/1782)	<.001
SAVR	16.7 (70/420)	4.3 (76/1782)	<.001
CABG	2.6 (11/420)	3.7 (66/1782)	.276
Other	9.3 (39/420)	2.4 (43/1782)	<.001
Hypertension	29.8 (125/420)	34.3 (612/1782)	.073
Hypercholesterolemia	12.4 (52/420)	14.8 (264/1782)	.201
Diabetes mellitus	9.3 (39/420)	12.2 (218/1782)	.091
Arterial disease	3.6 (15/420)	2.6 (47/1782)	.298
Peripheral	3.6 (15/420)	2.4 (42/1782)	.159
Carotid	0.5 (2/420)	0.3 (5/1782)	.522
Renal failure	2.6 (11/420)	2.3 (33/1782)	.312
Previous myocardial infarction	4.3 (18/420)	5.6 (99/1782)	.297
Previous revascularization	8.6 (36/420)	7.8 (139/1782)	.599
Previous PCI	6.4 (27/420)	5.1 (90/1782)	.257
Previous CABG	2.6 (11/420)	3.7 (66/1782)	.276
Previous decompensated heart failure	16.9 (71/420)	13.7 (245/1782)	.097
Left ventricular function			
Preserved	77.6 (287/370)	82.5 (1348/1633)	.026
Mildly reduced	7.6 (28/370)	6.3 (103/1633)	.376
Moderately reduced	9.2 (34/370)	8.3 (136/1633)	.592
Severely reduced	5.7 (21/370)	2.8 (46/1633)	.006
Atrial fibrillation	13.3 (56/420)	13.5 (241/1782)	.918
Previous neurologic event	10.5 (44/420)	8.0 (142/1782)	.096
CVA	4.8 (20/420)	3.5 (62/1782)	.212
TIA	7.1 (30/420)	5.1 (91/1782)	.099
COPD	8.3 (35/420)	11.5 (205/1782)	.061
Liver disease	1.4 (6/420)	0.2 (4/1782)	.001
History of malignancy	8.1 (34/420)	6.1 (109/1782)	.139
Urgency			
Elective	49.3 (173/351)	62.0 (975/1573)	<.001
Semi-elective	46.7 (164/351)	37.6 (591/1573)	.001
Urgent	4.0 (14/351)	0.4 (7/1573)	<.001
Logistic EuroSCORE	5.7 ± 6.2 (204)	5.8 ± 5.8 (970)	.740
Mechanical prosthesis	66.7 (280/420)	48.0 (855/1782)	<.001
Year of operation			
1987-1994	24.5 (103/420)	16.3 (290/1782)	<.001
1995-2001	23.3 (98/420)	25.4 (452/1782)	.387
2002-2008	26.7 (112/420)	28.2 (502/1782)	.536
2009-2015	25.5 (107/420)	30.2 (538/1782)	.056

Data are presented as % (n/N) and mean ± standard deviation or median (interquartile range). *AS*, Aortic valve stenosis *AR*, aortic regurgitation, *SAVR*, surgical aortic valve replacement; *CABG*, coronary artery bypass grafting; *PCI*, percutaneous coronary intervention; *CVA*, cerebrovascular accident; *TIA*, transient ischemic attack; *COPD*, chronic obstructive pulmonary disease; *EuroSCORE*, European System for Cardiac Operative Risk Evaluation.

### Revascularization After SAVR

The mean follow-up after SAVR was 17.2 years, with a total follow-up accumulating to 4541 patient-years. During follow-up, 24 patients underwent coronary revascularization, with 3 patients requiring a second and 1 patient requiring a third revascularization procedure. In the time-to-first event competing risk analysis with mortality, the rates of revascularization were 0.5%, 0.5%, 2.2%, 4.1%, 5.3%, and 6.9% at 30 days and 1, 5, 10, 15, and 20 years of follow-up, respectively (Figure 2). The mean time to the first revascularization was 8.9 ± 7.4 (range 0-26.9 years). The linearized rate of revascularization was 6.2 per 1000 patient-years.

### Characteristics of Revascularization

More patients underwent PCI than CABG, accounting for 64.2% of revascularization procedures (n = 18/28). Three patients (12.5%) needed urgent revascularization due to acute myocardial infarction (treated with PCI in all cases). Single-vessel disease was present in 16 patients (67%) and multivessel disease was present in 8 patients (33%). Four patients had lesions in both the left and right coronary artery. Characteristics of revascularization are displayed in Table 3.

**Table 3:** Characteristics of revascularization after SAVR

Patient	Date of SAVR	Revascularization after SAVR					Previous revascularization before SAVR		Subsequent revascularization(s)	
		Date	Urgency	Lesion	Modality	Details	Date	Modality	Date	Modality
#1	June 25, 1987	June 1, 1995	Elective	OM, IM	PCI				September 11, 1995	PCI
#2	August 12, 1987	December 20, 2007	Elective	OM, PD	CABG	SVG-OM-PD				
#3	May 18, 1988	June 24, 2003	Elective	LAD	CABG	SVG-LAD				
#4	June 3, 1988	November 21, 2003	Elective	RCA	PCI					
#5	September 1, 1988	August 4, 2015	Elective	LAD, LCx	PCI					
#6	March 21, 1989	November 4, 1994	Elective	RCA	PCI				January 29, 2001, and September 12, 2001	CABG and CABG
#7	July 25, 1990	March 29, 1993	Elective	LAD	CABG	LIMA-LAD				
#8	October 7, 1993	September 27, 2004	Elective	LAD	PCI				August 27, 2012	PCI
#9	November 9, 1993	March 10, 2015	Elective	LAD, RCA	PCI					
#10	July 1, 1998	August 3, 2012	Elective		CABG	SVG-RCA				
#11	August 7, 1998	June 30, 2015	Urgent	LAD, RCA	PCI					
#12	June 2, 2001	July 4, 2014	Elective	LAD	CABG	LIMA-LAD				
#13	November 28, 2002	September 3, 2009	Elective	LAD, IM, OM	CABG	LIMA-LAD SVG-IM-OM				
#14	January 31, 2003	December 6, 2005	Elective	RCA	PCI		October 30, 2002	PCI		
#15	December 20, 2004	October 26, 2010	Elective	OM	PCI					
#16	June 28, 2006	May 2, 2014	Urgent	SVG	PCI		May 2, 2000	CABG		
#17	October 31, 2008	December 7, 2012	Elective	RCA	PCI		January 19, 2004	PCI		
#18	November 4, 2008	August 27, 2012	Elective	RCA	PCI		September 27, 2004	PCI		
#19	May 13, 2009	December 31, 2015	Elective	LAD, LCx	PCI		May 20, 2003	PCI		
#20	December 2, 2011	January 9, 2013	Elective	OM	PCI		November 4, 2011	PCI		
#21	April 27, 2012	February 5, 2015	Urgent	LAD	PCI		July 17, 1997	CABG		
#22	October 5, 2012	March 11, 2015	Elective	LAD	CABG	LIMA-LAD				
#23	May 2, 2013	May 2, 2013	Elective	PD	CABG	SVG-PD				
#24	October 18, 2013	October 24, 2013	Elective	LCx	PCI					

*SAVR*, Surgical aortic valve replacement; *OM*, obtuse marginal artery; *IM*, intermediate artery; *PCI*, percutaneous coronary intervention; *PD*, posterior descending artery; *SVG*, saphenous vein graft; *LAD*, left anterior descending artery; *RCA*, right coronary artery; *LCx*, left circumflex artery; *CABG*, coronary artery bypass grafting; *LIMA*, left internal mammary artery.

### Subgroup Analysis and Predictors of Revascularization After SAVR

**Figure 3:**
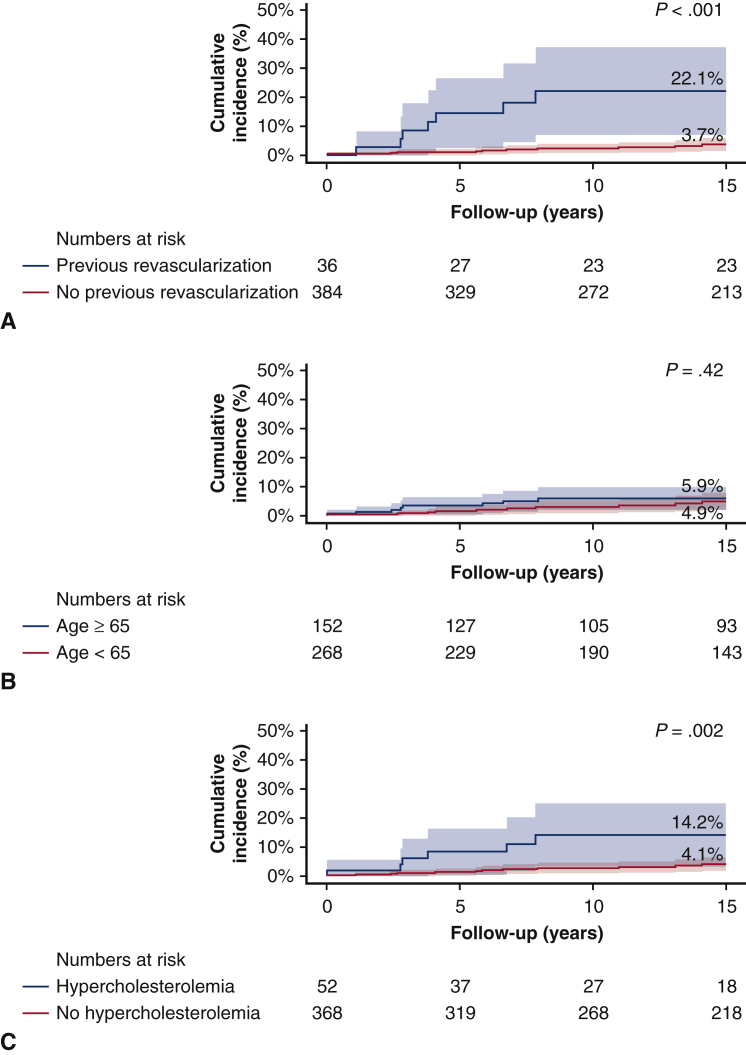
Revascularization after SAVR in various patient subgroups. Competing risk cumulative incidences of revascularization after SAVR in subgroups according to the following: (A) with and without previous revascularization. *Blue line* shows patients with no history of revascularization. *Red line* shows patients with a history of revascularization. (B) Age at SAVR younger or older than 65 years. *Blue line* shows patients aged 65 or older. *Red line* shows patients aged younger than 65 years. (C) With and without a history of hypercholesterolemia. *Blue line* shows patients with history of hypercholesterolemia. *Red line* shows patients without a history of hypercholesterolemia.

**Figure 4:**
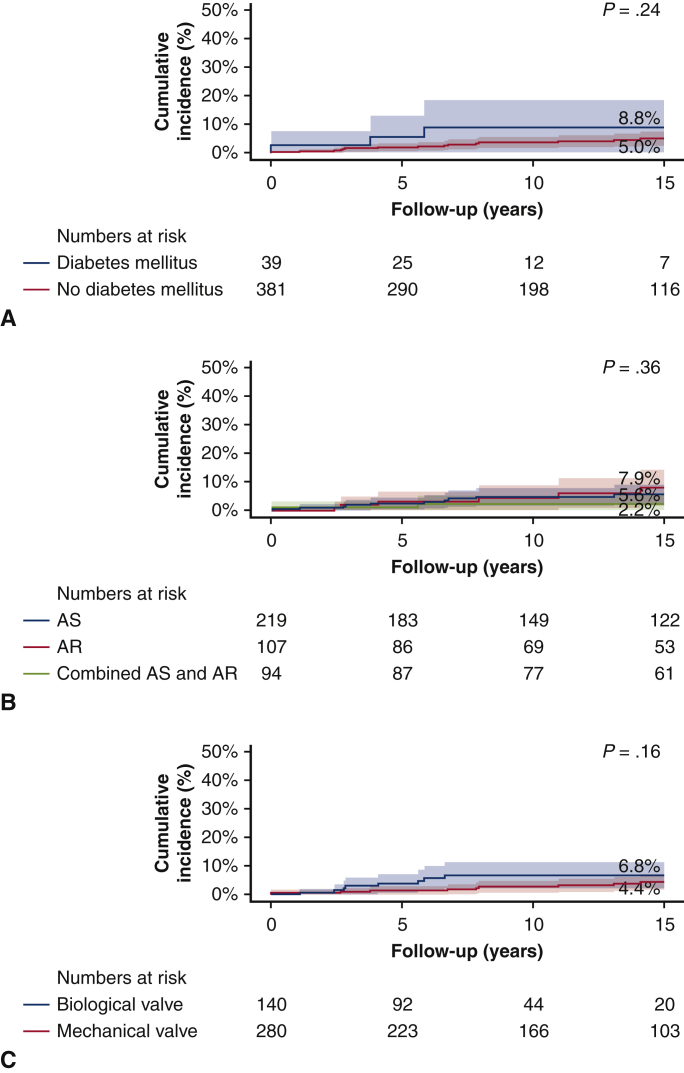
Revascularization after SAVR in various patient subgroups. Competing risk cumulative incidences of revascularization after SAVR in subgroups according to the following: (A) with and without a history of diabetes mellitus. *Blue line* shows patients with history of diabetes mellitus. *Red line* shows patients without a history of diabetes mellitus. (B) Primary indication for SAVR. *Blue line* shows patients undergoing SAVR for AS. *Red line* shows patients undergoing SAVR for AR. *Green line* shows patients undergoing SAVR for combined AS and AR. (C) Mechanical or biological prosthesis received. *Blue line* shows the use of a biological valve. *Red line* shows the use of a mechanical valve. *AS*, Aortic valve stenosis; *AR*, aortic regurgitation.

The incidence of revascularization at 15 years of follow-up was significantly greater in patients with previous revascularization than in patients without previous revascularization (22.1% vs 3.7%, *P* < .001), respectively. Further, the incidence of revascularization was greater in patients with hypercholesterolemia compared with patients without hypercholesterolemia (14.2% vs 4.1%, *P* = .002), respectively. There were no differences in revascularization rates during follow-up in subgroups according to age (4.9% for patients aged <65 vs 5.9% for patients aged ≥65, *P* = .42), diabetes mellitus (8.8% for patients with a history of diabetes mellitus vs 5.0% for no diabetes mellitus, *P* = .24), primary indication for SAVR (5.6% for AS vs 7.9% for aortic valve regurgitation vs 2.2% for combined disease, *P* = .36), or type of valve used (6.8% for biological vs 4.4% for mechanical, *P* = .16) (Figures 3 and 4).

### Factors Associated With Coronary Revascularization During Follow-up

Patients who underwent coronary revascularization during follow-up more often had hypercholesterolemia at baseline (8/24 vs 44/396, *P* = .001) and undergone revascularization before the index procedure (7/24 vs 29/396, *P* < .001) than patients that did not undergo revascularization during follow-up (Table 1). In multivariable analyses, the presence of revascularization, hypercholesterolemia, and diabetes mellitus before the index procedure were the only independent predictor of revascularization during follow-up (Table 4).

**Table 4:** Predictors of revascularization after SAVR

Characteristics	Univariable HR (95% CI); *P* value	Multivariable HR (95% CI); *P* value
Age	1.0 (1.0-1.1); *P* = .16	
Sex (female)	1.5 (0.6-3.4); *P* = .35	
Indication AS	1.1 (0.5-2.5); *P* = .79	
Indication AR	1.1 (0.4-2.7); *P* = .90	
Indication AS + AR	0.8 (0.3-2.1); *P* = .68	
Hypertension	1.2 (0.5-2.9); *P* = .68	
Hypercholesterolemia	5.0 (2.1-11.7); *P* < .001	3.4 (1.3-8.6); *P* = .010
Diabetes mellitus	3.2 (1.1-9.7); *P* = .037	2.1 (0.7-6.5); *P* = .214
Arterial disease	3.7 (0.9-15.9); *P* = .08	
Renal failure	3.9 (0.5-29.1); *P* = .19	
Previous MI	2.7 (0.6-11.7); *P* = .17	
Previous revascularization	8.2 (3.3-20.2); *P* < .001	6.6 (2.6-17.1); *P* < .001
Decompensated heart failure	1.8 (0.7-4.6); *P* = .20	
LVEF <50%	1.2 (0.4-3.6); *P* = .76	
Atrial fibrillation	1.0 (0.3-3.4); *P* = .97	
Previous stroke or TIA	0.0 (0.0-18.5); *P* = .31	
COPD	1.7 (0.4-7.3); *P* = .49	
Urgent SAVR vs non-urgent	1.6 (0.2-12.2); *P* = .64	
Log EuroSCORE	1.1 (1.0-1.1); *P* = .078	
Mechanical prosthesis	0.5 (0.2-1.3); *P* = .18	

*HR*, Hazard ratio; *CI*, confidence interval; *AS*, aortic valve stenosis; *AR*, aortic regurgitation; *MI*, myocardial infarction; *LVEF*, left ventricular ejection fraction; *TIA*, transient ischemic attack; *COPD*, chronic obstructive pulmonary disease; *SAVR*, surgical aortic valve replacement; *EuroSCORE*, European System for Cardiac Operative Risk Evaluation.

## Discussion

**Figure 5:**
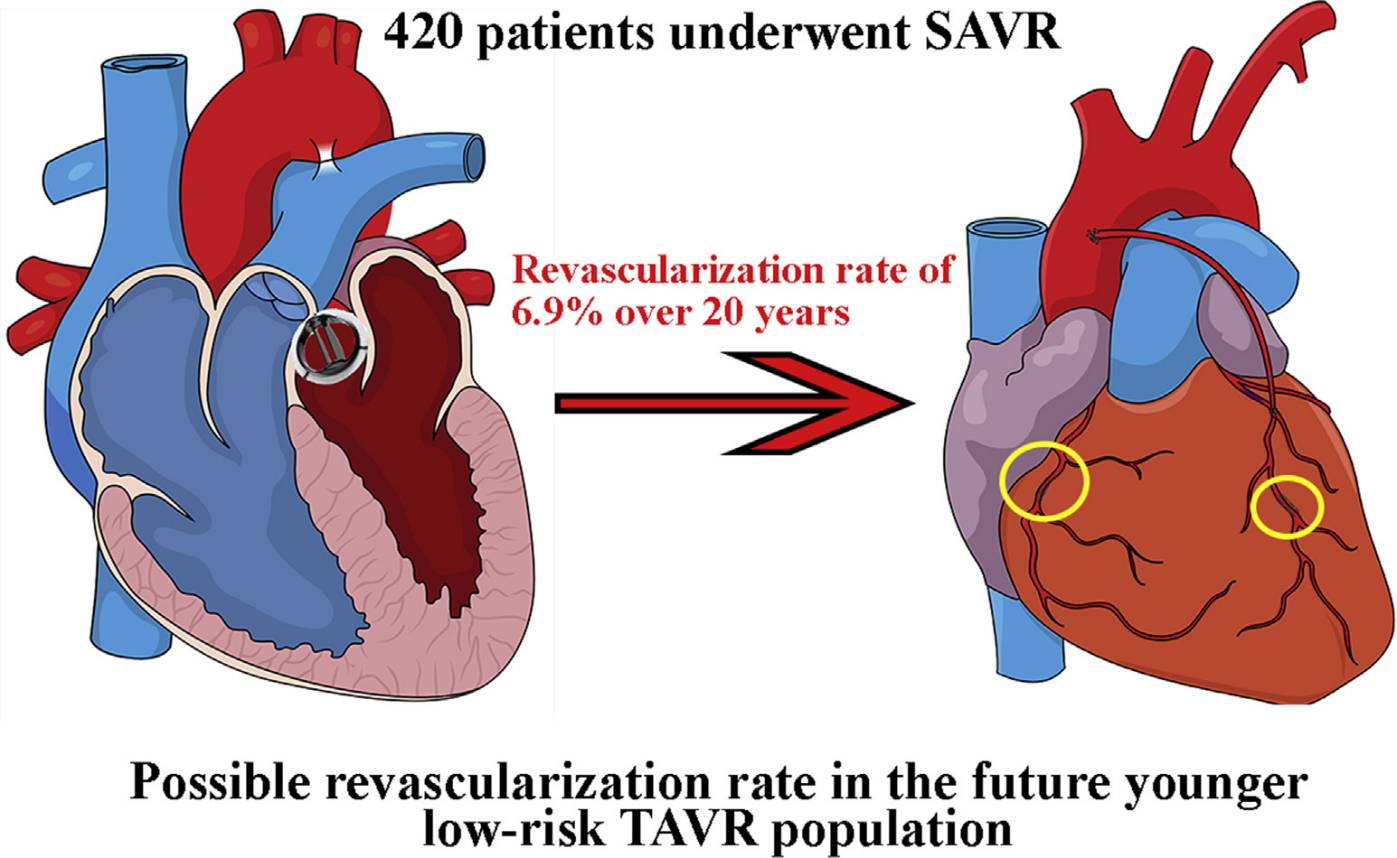
Cumulative competing risk incidence of revascularization presented as a graphical abstract. Competing risk cumulative incidence of coronary revascularization during 20-year after surgical aortic valve replacement. Coronary revascularization either done with coronary artery bypass grafting or percutaneous coronary intervention. Percutaneous coronary intervention is *encircled*. *SAVR*, Surgical aortic valve replacement; *TAVR*, transcatheter aortic valve replacement.

In this cohort of 420 patients who underwent isolated SAVR, 24 (5.7%) patients underwent a total of 28 revascularization procedures. The cumulative incidence of revascularization was 6.9% at 20-year follow-up, with a linearized rate of 6.2 per 1000 patient-years. In the current study, concomitant CABG was generally performed in patients with significant coronary stenosis. The risk of requiring coronary intervention during follow-up after SAVR in patients with no significant coronary stenosis at the time of intervention appears to be low as 6.9% at 20-year follow-up (Figure 5).

The incidence of revascularization was greater than that of the general population. Subgroup analyses showed that patients who had undergone previous revascularization before SAVR and patients with a history of hypercholesterolemia had significantly greater rates of revascularization during follow-up. Clearly patients with already established CAD, but nonsignificant at the time of SAVR, carry a risk of progression of CAD to a severity requiring intervention. Other risk factors of CAD, like hypertension and diabetes, were not associated with revascularization in our multivariable analysis, although this may be the result of a relatively low sample size in our study.

Of the patients who underwent revascularization, 16 patients had single-vessel disease and 8 patients 2-vessel disease. There were no patients with left main or 3-vessel disease. Considering the current guidelines for revascularization, the majority of patients would be referred for PCI on the basis of the complexity of coronary disease.^12^ Eight patients with more complex coronary disease underwent CABG during follow-up.

These data are important in the era of expanding indications for TAVR. Recently, 2 randomized controlled trials showed significant benefit of TAVR compared with SAVR in the low-risk population.^5^^,^^6^ Revascularization with PCI after TAVR can be associated with multiple technical challenges related to transcatheter heart valve platform, coronary access, with potential consequences of (1) damaging the prosthetic heart valve, (2) dissecting the coronary artery, (3) acute kidney injury related to increased contrast usage, and (4) an unsuccessful procedure.^13^ Because CAD is present in 40% to 75% of patients undergoing TAVR,^14^ algorithms on obtaining coronary access have already been developed from experiences during concomitant or staged TAVR and PCI procedures.^7^ The presence of CAD in the younger population undergoing TAVR is not well known, as studies mostly consist of elderly patients. Therefore, this study is the first to systematically assess the long-term rate of revascularization after aortic valve intervention in low-risk patients without CAD. Although our population consists exclusively of isolated SAVR procedures, it provides evidence on rates of revascularization that may be extrapolated to an overall TAVR population of low- to high-risk patients. Yet, literature also suggests that a proportion of patients might benefit from revascularization in the setting of acute coronary syndrome post-TAVR, and therefore greater incidences of revascularization could be expected in patients who initially would have been treated with medical therapy, when TAVR will expand toward the younger population.^15^

Of note, the mean age of our population was 57 years old as opposed to the current TAVR population with an advanced age, but a subgroup analysis according to age showed that the long-term rate of revascularization was comparable in patients younger or older or equal to 65 years. Expanding indication to lower-risk patients may have consequences for valve choice, given the younger age, and considering that coronary access is more challenging with a supra-annular TAVR than an intra-annular TAVR.^7^

### Limitations

This is a retrospective study that has inherent shortcomings related to data collection, changes in definitions of comorbidities, and patients being lost to follow-up. However, we included only patients who were followed after SAVR at our own outpatient clinic to minimize this risk. The multivariable analyses to identify predictors of revascularization may have been underpowered due to the small number of patients that needed a revascularization procedure and the unavailability of all known risk factors for coronary artery disease. Furthermore, although the decision was made not to include patients undergoing SAVR with concomitant CABG in this cohort, we did not have any information on the presence and degree of nonsignificant CAD that may increase the risk of coronary revascularization during follow-up as a result of progression of disease.

## Conclusions

In this retrospective analysis of patients who underwent isolated SAVR, the rate of requiring coronary revascularization at 20-year follow-up was relatively low. However, the rate was greater in patients who had undergone previous revascularization at the time of SAVR. These data provide some insights into requirements for coronary revascularization that may be relevant for the TAVR population. Future, larger studies are required on surgical and transcatheter cohorts to provide more insights into which patients are at particular risk of requiring coronary revascularization after aortic valve intervention.

### Conflict of Interest Statement

Dr van Mieghem has received institutional research grant support from 10.13039/100008497Boston Scientific, 10.13039/100004374Medtronic, and 10.13039/100000046Abbott and is an advisor to 10.13039/100004374Medtronic and 10.13039/100008497Boston Scientific. Dr Head is an employee of Medtronic, plc. Prof Kappetein is also an employee of Medtronic, plc. All other authors reported no conflicts of interest.

The *Journal* policy requires editors and reviewers to disclose conflicts of interest and to decline handling or reviewing manuscripts for which they may have a conflict of interest. The editors and reviewers of this article have no conflicts of interest.
